# Effect of a Herbal-Leucine mix on the IL-1β-induced cartilage degradation and inflammatory gene expression in human chondrocytes

**DOI:** 10.1186/1472-6882-11-66

**Published:** 2011-08-19

**Authors:** Nahid Akhtar, Mark JS Miller, Tariq M Haqqi

**Affiliations:** 1 Department of Medicine/Rheumatology, MetroHealth Medical Center, Case Western Reserve University, 2500 Metro Health Drive, Cleveland, Ohio 44109, USA; 2 MJSM: 2801 Summit Avenue, Plano 75074, Texas, USA

## Abstract

**Background:**

Conventional treatments for the articular diseases are often effective for symptom relief, but can also cause significant side effects and do not slow the progression of the disease. Several natural substances have been shown to be effective at relieving the symptoms of osteoarthritis (OA), and preliminary evidence suggests that some of these compounds may exert a favorable influence on the course of the disease. The objective of this study was to investigate the anti-inflammatory/chondroprotective potential of a Herbal and amino acid mixture containing extract of the *Uncaria *tomentosa, *Boswellia spp*., *Lepidium meyenii and L-Leucine *on the IL-1β-induced production of nitric oxide (NO), glycosaminoglycan (GAG), matrix metalloproteinases (MMPs), aggrecan (ACAN) and type II collagen (COL2A1) in human OA chondrocytes and OA cartilage explants.

**Methods:**

Primary OA chondrocytes or OA cartilage explants were pretreated with Herbal-*Leucine *mixture (HLM, 1-10 μg/ml) and then stimulated with IL-1β (5 ng/ml). Effect of HLM on IL-1β-induced gene expression of iNOS, MMP-9, MMP-13, ACAN and COL2A1 was verified by real time-PCR. Estimation of NO and GAG release in culture supernatant was done using commercially available kits.

**Results:**

HLM tested in these *in vitro *studies was found to be an effective anti-inflammatory agent, as evidenced by strong inhibition of iNOS, MMP-9 and MMP-13 expression and NO production in IL-1β-stimulated OA chondrocytes (p < 0.05). Supporting these gene expression results, IL-1β-induced cartilage matrix breakdown, as evidenced by GAG release from cartilage explants, was also significantly blocked (p < 0.05). Moreover, in the presence of herbal-*Leucine *mixture (HLM) up-regulation of ACAN and COL2A1 expression in IL-1β-stimulated OA chondrocytes was also noted (p < 0.05). The inhibitory effects of HLM were mediated by inhibiting the activation of nuclear factor (NF)-kB in human OA chondrocytes in presence of IL-1β.

**Conclusion:**

Our data suggests that HLM could be chondroprotective and anti-inflammatory agent in arthritis, switching chondrocyte gene expression from catabolic direction towards anabolic and regenerative, and consequently this approach may be potentially useful as a new adjunct therapeutic/preventive agent for OA or injury recovery.

## Background

Osteoarthritis (OA) is the most common form of joint disease that evolves from local inflammatory disease to a chronic process with variable degree of inflammation and degeneration of articular cartilage that ultimately results in exposure of underlying bone, pain and disability [1]. The aggregating cartilage proteoglycan, aggrecan (ACAN), along with the type II collagen (COL2A1) provide the robust mechanical properties to the cartilage in a healthy joint. The progressive degeneration of articular cartilage involves depletion of ACAN and the deregulation of matrix components, such as proteoglycan and collagen [2]. Enzymatic cleavage by matrix metalloproteinases (MMPs) is thought to be involved in the destruction of articular cartilage, so the high expression of MMP-13/collagenase-3 and MMP-9/gelatinase B detected in the pathologic synovium and cartilage samples is believed to contribute to OA pathogenesis [3,4]. IL-1β is implicated in the degeneration of articular cartilage due to its induction of proteoglycan loss and matrix degradation [5]. Elevated levels of IL-1 occur in the synovial fluid and cartilage tissue of patients with OA [6], implying a role in disease pathogenesis. IL-1 receptor antagonist, a natural competitor of IL-1, suppresses cartilage loss, further supporting the role of IL-1 in cartilage breakdown [7]. Both IL-1 and mechanical loading of cartilage increase nitric oxide (NO) production [8,9] by up-regulating the expression of inducible nitric oxide synthase gene (iNOS). NOS inhibitors inhibit the progression of OA in the experimental animal models [10], and the joint pathology is significantly inhibited in the collagen-induced arthritis model in NOS deficient mice [11]. Recent exceptional growth in human exposure to natural herbs used as traditional medicine has lead to a resurgence of scientific interest in their biological effects. Use of traditional medicinal plants for the treatment of OA has gained popularity because they are purported to show clinical efficacy with minimal side effects compared to mainstream treatment. Additionally, medicinal plants are often cheaper, locally available and easily consumable.

The *Boswellia *spp., which is native to Ethiopia, Somalia, India, and the Arabic peninsula, produce a gum resin that is known as olibanum (frankincense). The resin of *Boswellia carteri *and *Boswellia serrata *has been used for the treatment of rheumatoid arthritis and other inflammatory diseases in the traditional medicine system in many countries [12,13]. Besides their renowned anti-inflammatory activity, Boswellic acids have been extensively investigated with respect to their chemopreventive effects [14,15].

*Lepidium meyenii *(Maca) is an Andean plant of the Brassica (mustard) family. Preparations from maca root have been reported to be antioxidant [16,17] and improve sexual function [18]. We recently observed that Maca had chondroprotective properties in human cartilage [19]. This action was mediated in part through an up-regulation of gene expression of the anabolic growth factor IGF-1 even in the presence of the inflammatory cytokine IL-1. This effect on preventing cartilage breakdown and maintain balance of genetic resources governing catabolism and anabolism in human cartilage was enhanced when maca was combined with another South American medicinal plant Cat's claw (*Uncaria guianensis*). Follow up clinical studies in subjects with osteoarthritis of the knee indicated that this combination of cat's claw and maca was effective in alleviating arthritis symptoms in 92% of subjects [20]. Cat's claw is an Amazonian vine whose bark is used to make a decoction that has traditional use in managing inflammation, including joint health. This action has been supported by clinical studies in osteoarthritis [21] and attributed to its powerful antioxidant actions and its ability to inhibit the transcriptional factor NF-kB [17,22,23].

*Leucine *is a branch-chained amino acid that serves not only as a substrate for protein synthesis, but also as a nutrient signal to stimulate protein synthesis. The stimulation of protein synthesis achieved by *Leucine *supplementation occurs by the activation of translation initiation factors through the mTOR signaling pathway [24,25]. Reports state that *Leucine *supplementation can be used as an adjunct in the nutritional management for full protein delivery [26]. Joint health can be influenced by the supporting musculature and so approaches that enhance muscle mass and performance (nutritional and exercise) can be helpful in maintaining optimal joint health. However, to our knowledge little is known about the effects of *Leucine *on human chondrocytes.

The reason for using herbs in combination is that herbs have chemicals components which can bring strong beneficial effects. This also helps body to manage potentially undesirable effects of anyone/each herb in combination/formulation plays in a curative or pacifying role. It is therefore preferable to use herbal combination instead of depending on a single herb. However, the effect of Herbal-*Leucine *mixture (HLM) used in this study (contains three natural products and an amino acid: *Uncaria tomentosa, Boswellia spp*., *Lepidium meyenii and L-Leucine*) has not been studied in OA chondrocytes in relation to its chondroprotective/anti-inflammatory potential. We therefore studied the effect of HLM on the expression of key molecules believed to be involved in OA pathogenesis such as NO, COL2A1, ACAN, glycosaminoglycans (GAGs) and MMPs in human OA chondrocytes and cartilage explants, stimulated with IL-1β. We also investigated the effect of HLM on the activation of NF-kB in chondrocytes, which is a master transcription factor involved in up-regulation of many of these inflammatory genes during OA pathogenesis.

## Methods

### Cell isolation and human chondrocytes culture

The protocol was approved and permission to use discarded and de-identified human tissue was obtained from the Institutional Review Board, MetroHealth Medical Centre (IRB09-01330), prior to the initiation of the studies. These studies were approved as "exempt" and that no informed consent was required. OA was diagnosed according to the American College of Rheumatology criteria [27,28]. Human chondrocytes were prepared by enzymatic digestion of cartilage obtained from nine osteoarthritis (OA) patients (mean age, 60.6 ± 4.7 years) who underwent knee arthroplasty at the MetroHealth Medical Centre, Cleveland, OH. Specimens that included the full thickness cartilage and subchondral bone were washed with sterile PBS and the macroscopic cartilage degeneration was determined by staining with India ink [29]. Portions of the cartilage with smooth articular surface were used to prepare chondrocytes by the pronase and collagenase enzymatic digestion (OA chondrocytes) as previously described [30,31]. Primary OA chondrocytes were plated at the seeding density of 1 × 10^6 ^cell/35 mm dish and at 80% confluence were used for all the studies described here.

### Preparation of Herbal-Leucine mixture (HLM) and treatment of chondrocytes with IL-1β

Herbal-*Leucine *mixture (HLM) was prepared by mixing extracts of *Uncaria tomentosa *(Cats Claw), *Boswellia spp*., *Lepidium meyenii *(maca) *and pharmaceutical grade L-Leucine *(Ajinimoto USA Inc, Fort Lee, NJ). The final mixture is a commercial product called FlexSure, (Vital g-Netics, Quitman, TX) and the recommended dose contains 1000 mg *Lepidium meyenii*, 300 mg *Uncaria tomentosa*, 200 mg *Boswelia serrata*, 700 mg *L-Leucine*. OA chondrocytes at 80% confluence were serum starved in DMEM starving medium for 12-16 h and were pretreated with the HLM (1-10 μg/ml) for 2 h and were then stimulated with IL-1β (5 ng/ml) (R&D system, St. Paul, MN) for 6 or 24 h. An inhibitor of nuclear factor-kB (NF-kB), MG132 (100 μM) was used as a positive control to study the effect of HLM on MMP-9, MMP-13 production and NF-kB inhibition. OA chondrocytes cultured without IL-1β served as controls. Culture supernatants were used to estimate total nitric oxide production.

### Cell viability assay

OA chondrocytes were treated with the HLM (1 and 5 μg/ml) for 24 h and the cytotoxic effect of the herbal mixture was evaluated by lactate dehydrogenase (LDH) activity using CytoTox96^® ^Non-Radioactive Cytotoxicity Assay kit (Promega, Madison, WI). LDH is a soluble cytosolic enzyme that is released into the culture medium following loss of membrane integrity resulting from either apoptosis or necrosis. LDH activity, therefore, can be used as an indicator of cell membrane integrity and serves as a general means to assess cytotoxicity resulting from chemical compounds or environmental toxic factors. Kit measures LDH activity present in the culture medium using a coupled two-step reaction. In the first step, LDH catalyzes the reduction of NAD^+ ^to NADH and H^+ ^by oxidation of lactate to pyruvate. In the second step of the reaction, diaphorase uses the newly-formed NADH and H^+ ^to catalyze the reduction of a tetrazolium salt (INT) to highly-colored formazan which absorbs strongly at 490 nm.

### Nitrite assay

The interaction of NO in a system is measured by the determination of total nitrate and nitrite concentrations in the samples. Chondrocytes were pretreated for 2 h with HLM (1 and 5 μg/ml) and then stimulated or not stimulated with IL-1β (5 ng/ml) for 24 h. Total nitric oxide assay kit (Thermo Scientific, Rockford, IL) was used for the assessment effects of IL-1β induced nitric oxide production in culture supernatants. The kit uses the enzyme nitrate reductase to convert the nitrate to nitrite. Nitrite is then detected as a colored azo dye product the Griess reaction that absorbs visible light at 540 nm.

### Quantitative real-time-PCR (RT-PCR)

Real time quantitative-PCR was used to quantify the mRNA expression of matrix metalloproteinase-9 (MMP-9, assay ID Hs00234579), MMP-13 (assay ID Hs00233992), Aggrecan (ACAN, assay ID Hs00153936), Type-II collagen (COL2A1, assay ID 00264051), and inducble nitric oxide synthase (iNOS, assay ID Hs01075526) using TaqMan Gene expression assays (Applied Biosystems, Foster city, CA). Total RNA was isolated from OA chondrocytes by Trizol reagent (Invitrogen, Carlsbad, CA) according to the manufacturer's instruction. First-strand cDNA was synthesized using 500 ng of total RNA and the QuantiTect Reverse Transcription kit (Qiagen, Valencia, CA). Quantitative PCR was performed in 20 μl reactions containing 2 μl of 10-times diluted RT product, 10 μl of 2X TaqMan Universal Master Mix (Applied Biosystems, Foster city, CA), 0.2 μM TaqMan probe and 0.9 μM forward and reverse primers. Reaction mixtures were incubated at 95°C for 10 min, followed by 40 cycles of 95°C for 30 sec and 60°C for 1 min. Expression of GAPDH was used as endogenous control. A threshold cycle (C_t_) was observed in exponential phase of amplification and quantification of relative expression levels was determined by ΔΔC_t _method [32].

### Enzyme linked immune-sorbant assays (ELISA)

Human chondrocytes were pretreated for 2 h with HLM (1-10 μg/ml) and then stimulated or not stimulated with IL-1β (5 ng/ml) for 24 h. The effect of IL-1β and/or HLM on the level of MMP-9 and MMP-13 secreted by chondrocytes in the culture medium was further confirmed by sandwich ELISAs (R&D systems, Minneapolis, MN) and all the assays were performed according to the manufacturer's instructions. Limit of detection for MMP-9 and MMP-13, was > 0.156 ng/ml, and 7.7 pg/ml respectively.

### Preparation and treatment of cartilage explants

Full thickness cartilage slices were dissected from the unaffected cartilage using sterile scalpel blade (Feather Safety Razor Co. Osaka, Japan). Five cartilage pieces (26.7 mg ± 1.3) were transferred to each well of a 24-well, flat bottom plate (Falcon, NJ, USA) containing Dulbecco's modified Eagle's medium (DMEM) supplemented with antibiotics and 10% fetal bovine serum (FBS). After 24 h cultures, the medium was changed to starving medium (DMEM) and explants were starved for 12-16 h. The cartilage explants were treated with IL-1β alone or with the HLM (1 and 5 μg/ml) or HLM alone for 7 days with the replacement of reagents every two days without medium change. Culture supernatants were used for determination of GAG release.

### Determination of glycosaminoglycans (GAGs)

Level of sulfated GAGs released in the medium was measured by a metachromatic dye 1, 9-dimethylmethylene blue (DMMB) using proteoglycan detection kit (Astarte Biologics, WA). Briefly, 100 μl of DMMB reagent was added to 100 μl of culture supernatant. The GAG-dye complex results in absorption spectrum shift, which can be measured at 525 nm. Values were derived from standard curve using different concentrations of chondroitin sulfate and the results are expressed as microgram GAG/gram of cartilage.

### Transient transfection and luciferase activity assay

To study the effect of HLM on the IL-1β-induced activation of NF-kB, human chondrocytes were transiently transfected with p-NF-kB-Luc reporter plasmid (Agilent Technologies, Santa Clara, CA). Briefly, OA chondrocytes were seeded at 1 × 10^6^/well in 6 well plates and were transfected with p-NF-kB-Luc reporter plasmid using Lipofectamine reagent (Invitrogen, Carlsbad, CA) according to the instruction of the manufacturer. Transfected cells were pretreated with HLM (1-10 μg/ml) and/or stimulated with IL-1β for 24 h. Cells were washed with ice-cold PBS, lysed using passive lysis buffer and the luciferase activity was determined using a commercially available kit according to the instructions of the manufacturer (Luciferase assay kit, Promega, Madison, WI).

### Statistical analysis

All experiments were performed three times using independent samples. Values shown are mean ± SE unless stated otherwise. Comparisons were performed using Origin 8.1 software package (one paired two tailed *t*-test with one way ANOVA and Tukey's post-hoc analysis) and *p *< 0.05 was considered significant.

## Results

### Effects of Herbal-Lucine mixture (HLM) on the chondrocyte viability

**Figure 1:**
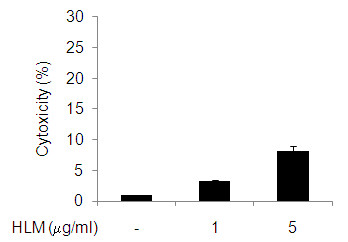
**Chondrocyte viability after treatment with HLM (*Uncaria tomentosa*., *Boswellia spp*., *Lepidium meyenii and L-Leucine*)**. Human OA chondrocytes were incubated with 1 and 5 μg/ml of HLM for 24 h and viability was determined using release of lactate dehydrogenase (LDH) in culture supernatant. Values given are mean ± SD of 4 experiments.

LDH cytotoxicity assay showed that the HLM used did not reduce the chondrocytes viability significantly (p > 0.05). Ninety-two percent of chondrocytes treated with the HLM up to 5 μg/ml for 24 h were found to be viable (Figure 1).

### Inhibition of inducible nitric oxide synthase (iNOS) and nitric oxide (NO) production by the Herbal-Leucine mixture in IL-1β-stimulated human chondrocytes

**Figure 2:**
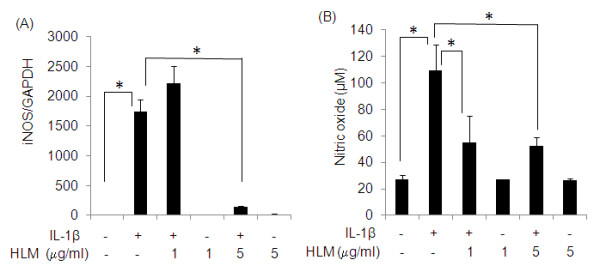
**Effect of HLM (*Uncaria tomentosa*., *Boswellia spp*., *Lepidium meyenii and L-Leucine*) on the IL-1β-induced gene expression of inducible nitric oxide synthase (iNOS) (A) and on the production of nitric oxide (NO) (B) in human OA chondrocytes**. Chondrocytes were pretreated for 2 h with HLM (1-5 μg/ml) and then stimulated or not stimulated with IL-1β (5 ng/ml) for 24 h. Relative gene expression of iNOS was determined by Real-time PCR using GAPDH as endogenous control and compared with un-stimulated control. Corresponding culture supernatants were analyzed for total NO levels. Values represent Mean ± SE of three different experiments run in duplicate. * p < 0.05.

The effect of the HLM on IL-1β-induced iNOS expression and NO production is shown in Figure 2A and 2B. Human OA chondrocytes were pretreated with HLM (1 and 5 μg/ml) for 2 h and then stimulated or not stimulated with IL-1β (5 ng/ml) for 24 h. Stimulation with IL-1β significantly increased the expression of iNOS (1740-fold; p < 0.05) and production of NO (4-fold; p < 0.05) respectively, compared to un-stimulated control. Importantly, cytokine-stimulated increase in iNOS mRNA levels was significantly inhibited (92%; p < 0.05) by the pretreatment of OA chondrocytes with HLM (5 μg/ml) compared to IL-1β-stimulated OA chondrocytes. Similarly, there was a significant down regulation in NO production (50% and 52%; p < 0.05) in IL-1β-stimulated OA chondrocytes cultures that were pretreated with the HLM at 1 and 5 μg/ml, respectively.

### Down-regulation of IL-1β-induced MMP-9 and MMP-13 mRNA and protein expression in human chondrocytes

**Figure 3:**
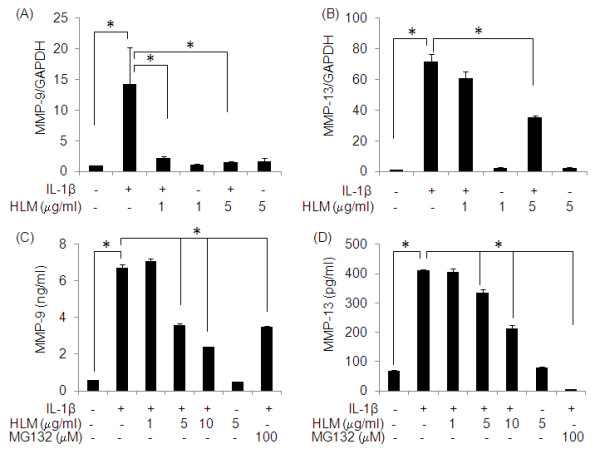
**Effect of HLM (*Uncaria tomentosa*., *Boswellia spp*., *Lepidium meyenii and L-Leucine*) on the IL-1β-induced expression of MMP-9 and MMP-13 in IL-1β-stimulated OA chondrocytes**. Chondrocytes were pretreated for 2 h with HLM (1-10 μg/ml) and then stimulated or not stimulated with IL-1β (5 ng/ml) for 6 h for MMP-9 mRNA (A) and 24 h for MMP-13 mRNA(B). mRNA expression was analyzed by Real time-PCR. Relative gene expression was normalized to GAPDH and compared with un-stimulated control. Levels of MMP-9 (C) and MMP-13 (D) in culture supernatants were quantified by sandwich ELISA at 24 h. Value represents Mean ± SD of three different experiments runs in duplicate. * p < 0.05.

Next we investigated the impact of treatment with HLM on the IL-1β-induced MMP-9 and MMP-13 mRNA and protein expression in human OA chondrocytes. As shown in Figure 3A and 3B, IL-1β-stimulation of human OA chondrocytes resulted in significant up-regulation of MMP-9 (14-fold) and MMP-13 (71-fold) mRNA expression (p < 0.05). The treatment of chondrocytes with the HLM (1 and 5 μg/ml) showed 84-89% decrease in the level of MMP-9 mRNA expression (p < 0.05), compared to OA chondrocytes not treated with the HLM but were stimulated with IL-1β alone. To determine whether this inhibition of gene expression also influenced the MMP-9 protein production, culture supernatants were assayed for MMP-9 protein using a sandwich ELISA. A significant increase in secreted MMP-9 in the culture supernatant (11.7-fold) was found when OA chondrocytes were stimulated with IL-1β (Figure 3C; p < 0.05). However, pretreatment of chondrocytes with HLM significantly inhibited the MMP-9 production in IL-1β-stimulated OA chondrocytes (64%, Figure 3C; p < 0.05). This inhibition in MMP-9 production was also noted when chondrocytes were pre-treated with MG132, a known NF-kB inhibitor, which inhibited the MMP-9 production by 47% (Figure 3C; p < 0.05). Also pretreatment with HLM inhibited the IL-1β-induced MMP-13 mRNA expression by 51% at 5 μg/ml (Figure 3B; p < 0.05). OA chondrocytes stimulated with IL-1β alone showed 504% increase in MMP-13 protein production, which was dose dependently inhibited (upto 49%) by HLM (Figure 3D; p < 0.05). Interestingly, MG132 pretreatment also inhibited 99% of MMP-13 production in OA chondrocytes.

### Effect of Herbal-Leucine mixture on IL-1β-induced inhibition of ACAN and COL2A1 mRNA expression in human chondrocytes

**Figure 4:**
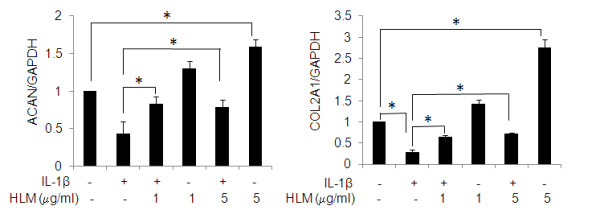
**Effect of HLM (*Uncaria tomentosa*., *Boswellia spp*., *Lepidium meyenii and L-Leucine*) on the IL-1β-induced gene expression of ACAN and COL2A1 in IL-1β-stimulated OA chondrocytes**. Chondrocytes were pretreated for 2 h with HLM (1 and 5 μg/ml) and then stimulated or not stimulated with IL-1β (5 ng/ml) for 24 h, expression of ACAN (A) and COL2A1 (B) was analyzed by Real time-PCR. Relative gene expression was normalized to GAPDH and compared with un-stimulated control. Value represents Mean ± SD of three different experiments run in duplicate. * p < 0.05.

We explored if the HLM affected the ACAN and COL2A1 gene expression in chondrocytes stimulated with IL-1β or not stimulated but treated with HLM alone (Figure 4A and 4B). HLM alone treatment produced a significant increase in ACAN (50%) and COL2A1 (63%) mRNA expression at 5 μg/ml concentration in OA chondrocyte compared to un-stimulated control chondrocytes (p < 0.05). IL-1β-stimulation resulted in significant down regulation of ACAN (2.5-fold) and COL2A1 (5-fold) expression in OA chondrocytes. However, pretreatment of HLM (1 and 5 μg/ml) significantly inhibited the IL-1β-mediated down regulation of ACAN (49%; p < 0.05) and COL2A1 (56-62%; p < 0.05), compared to IL-1β alone stimulated OA chondrocytes.

### Inhibition of IL-1β-induced cartilage matrix degradation

**Figure 5:**
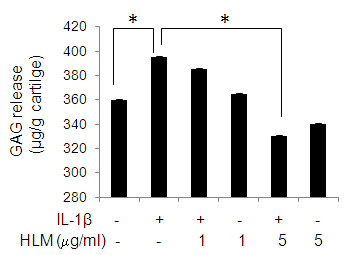
**Cartilage matrix status**. HLM inhibited the IL-1β-induced release of GAG from human cartilage explants *in vitro*. Cartilage pieces (26.7 mg ± 1.3) were incubated with medium alone or medium containing either IL-1β alone or in combination with HLM (1 and 5 μg/ml) for 7 days. Total GAG release from cartilage explants in culture supernatants was quantified by using Proteoglycan detection kit and values are derived from standard curve. Value represents Mean ± SD of three different experiments repeated in duplicate. * p < 0.05.

The effect of HLM on the IL-1β-induced cartilage matrix degradation is shown in Figure 5. Treatment with IL-1β-induced cartilage matrix degradation was increased by 9% in culture medium from cartilage explants, compared to un-stimulated control cartilage explants (Figure 5). However, the IL-1β-induced release of GAG was significantly inhibited by the HLM (16%; p < 0.05), compared to IL-1β-stimulated OA cartilage explants. These results provide support that HLM may to be an effective agent for blocking the IL-1β-induced release of GAGs from human cartilage, at least *in vitro*.

### Effect of HLM on NF-kB activation

**Figure 6:**
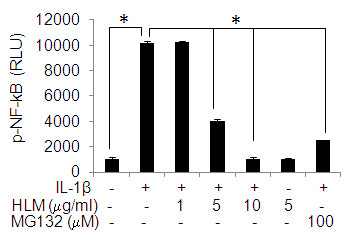
**Effect of HLM (*Uncaria tomentosa*., *Boswellia spp*., *Lepidium meyenii and L-Leucine*) on the IL-1β-induced activation of NF-kB in IL-1β-stimulated OA chondrocytes**. Chondrocytes were transfected with NF-kB luciferase plasmid and the NF-kB dependent transcriptional activity was determined by luciferase assay using commercially available kit (Promega). Value represents Mean ± SD of three different experiments repeated in duplicate. * p < 0.05.

To determine directly whether HLM inhibit activation of NF-kB in IL-1β-stimulated OA chondrocytes, we used NF-kB-dependent gene reporter assay. OA chondrocytes were transiently transfected with pNF-kB-Luc reporter plasmid and then stimulated with IL-1β. Stimulation of chondrocytes with IL-1β resulted in robust luciferase activity (Figure 6; p < 0.05). Interestingly, this luciferase activity was significantly reduced in OA chondrocytes treated with HLM prior to stimulation with IL-1β (Figure 6; p < 0.05). Similar inhibition of luciferase activity was observed when chondrocytes were pretreated with MG132. Taken together these data indicate that HLM was a potent suppressor of NF-kB activity in OA chondrocytes.

## Discussion

Osteoarthritis (OA) is a heterogeneous, complex joint pathology of unknown etiology. Apart from the surgical measures, treatment of OA has generally been aimed to alleviating major complaints, such as pain, swelling, and muscle tightness and thus resulting in improved mobility [33]. Current treatment options include Non-steroidal Anti-Inflammatory Drugs (NSAIDs) including cyclooxygenase-2 (COX-2) inhibitors (rofecoxib) and others for pain relief but fail to block the progression of the disease [34]. Unfortunately, these agents are also associated with gastrointestinal, cardiovascular and other risks and do not abrogate the loss of cartilage matrix [33,34]. Nutraceuticals derived from herbs, spices and medicinal plants have long been used in the traditional medicine system of many nations including India and China, and have been shown to be effective as non-steroidal anti-inflammatory drugs at relieving the symptoms of OA, and preliminary evidence suggests that some of these compounds may exert a favorable influence on the course of the disease. In this study, we investigated the anti-inflammatory/chondroprotective potential of a Herbal-*Leucine *mixture (HLM) of four natural products: *Uncaria tomentosa*., *Boswellia spp*., *Lepidium meyenii and L-Leucine *on the production of key molecules released during chronic inflammatory events such as nitric oxide (NO), glycosaminoglycan (GAG), matrix metalloproteinases (MMPs), aggracan (ACAN) and type II collagen (COL2A1) by human chondrocytes and cartilage in the presence of IL-1β. We have also studied the effect of HLM on the IL-1β-induced NF-kB activation in these cells.

*Uncaria *has been found to inhibit lipopolysaccharide (LPS)-induced iNOS gene expression, nitrate formation, cell death, PGE_2 _and TNFα production and the activation of NF-kB [23,35]. Both species of *Uncaria *(*U. tomentosa *and *U. guianensis*) were reported to be a strong free radical scavenger [17,22]. The available animal toxicological data did not indicate any severe toxicity by the oral intake of *Uncaria *[21,36]. Additionally, recent article supports the use of *Uncaria spp*. alone or in combination with other medicinal herbs for OA management [20,37].

A randomized clinical trial of multiplant Ayurvedic drugs containing *Boswellia Serrata *as one of the component demonstrated the potential efficacy and safety in the symptomatic treatment of knee OA over 32 weeks of therapy [38]. Another trial of a herbomineral formulation containing *Boswellia serrata *as one of the components also showed significant reduction in severity of pain and disability score [39]. Further, Boswellic acids have been reported as inhibitors of 5-lipoxygenase, the key enzyme for leukotriene biosynthesis in inflammatory disorders [40,41] and human leukocyte elastase [12]. Thus its use may be beneficial in inhibiting the severity of OA.

Although, the *Lepidium meyenii *root preparations have been reported to improve sexual function [18] and an antioxidant status [16] but, the role of *Lepidium meyenii *alone in OA management has not been investigated as yet. However, we noted that *Lepidium meyenii *had chondroprotective effects in human cartilage explants in a similar experimental protocol as this report [19] and as a component of an effective approach to the management of OA when administered in combination with *Uncaria guianensis *[20]. *L-Leucine *has clinical support for its benefits in exercise and sports performance, which indirectly can improve joint health or delay progressive nature of osteoarthritis [42,43]. However, a direct action of *L-Leucine *on arthritic processes is not supported in the literature. Based on the results of this study it may be worthy of future investigation.

Our results on HLM cytotoxicity shows that HLM had no significant effect on the chondrocytes viability at the concentrations used in 24 h cultures. Also, the effect of HLM on IL-1β-induced NO production are interesting as inhibition of NO has been associated with the reduced pain, inflammation, proteoglycan loss in human OA patients and in animal models of arthritis [10,11,44]. Importantly, our results showed that HLM significantly inhibited the iNOS expression and NO production in IL-1β-stimulated OA chondrocytes.

The MMPs are a family of 23 enzymes in humans which facilitate ECM turnover and breakdown under normal and disease conditions [3]. Pro-inflammatory cytokine IL-1β-induced MMP-13 production by chondrocyte is the foremost collagenase in OA pathogenesis. Several other members including MMP-9 have been localized to cartilage or synovium in the arthritis patients [3]. Screening of MMPs specific inhibitors has gained attention recently but due to their monomodal nature, lack of specificity and greater side effects there is a need to develop therapeutic strategies focusing on prophylactic agents [45].

Our study showed that HLM can potently down regulate the IL-1β mediated induction of MMP-13 and MMP-9 mRNA and protein expression in human OA chondrocytes *in vitro*. It is important to note that cartilage explants reflect the cellular responses of only one cell type, the chondrocyte, as cartilage is a vascular and comprised of chondrocytes and matrix only. Thus, our results suggest that HLM may diminish MMP-mediated degeneration of cartilage in OA through its effects on human chondrocytes. A recent study has shown that Aflapin a novel synergistic composition derived from *Boswellia serrata*, inhibit MMP-3 in a controlled clinical study [46]. *Boswellia frereana *extracts has also been reported to inhibit IL-1β and oncostatin M induced MMP-9 and MMP-13 expression in cartilage explants cultures [47]. Similarly, *Uncaria sinensis *has also shown significant inhibition against MMP-2 and -9 activities in vascular smooth muscle cells [48]. Thus the observed beneficial anti-inflammatory effects of HLM may be due to *Boswellia *and *Uncaria *combination present in HLM.

A consequence of OA is the pro-inflammatory cytokine IL-1β-induced down-regulation of cartilage anabolic factors such as ACAN, proteoglycan and COL2A1 [6]. Our results also showed that HLM has the potential to inhibit IL-1β-induced GAG release from cartilage explants. Beside this, HLM alone or in the presence of IL-1β has up-regulated the COL2A1 and ACAN expression in OA chondrocytes. Aggrecan is degraded predominantly by aggrecanases and MMPs [3]. The observed inhibition of GAG release by HLM may possibly be due to MMPs and NO inhibition by HLM. Moreover, induction of COL2A1 and ACAN expression by HLM suggests that this could intercept the process of cartilage degeneration during the pathogenesis of OA. Whether it can extend these benefits to building the mass of cartilage matrix beyond existing downsized levels needs further investigations.

The transcription factor NF-kB is a redox-sensitive transcription factor that regulates the expression of genes involved in osteoarthritis [49]. Because the suppression of NF-kB has been linked with anti-inflammatory activity, we postulated that HLM mediated inhibitory effects on the inflammatory genes expression discovered in this study could be at least in part through the suppression of NF-kB activity. The data indicated that HLM attenuates the IL-1β-induced activation of NF-kB in OA chondrocytes.

## Conclusions

In conclusion, we demonstrated that HLM inhibits the pro-inflammatory cytokine IL-1β-stimulated expression of major proteases (MMP-9 and MMP13), NO and GAG release associated with cartilage degradation. We also showed cartilage protective ability of HLM by up-regulating cartilage anabolic factors like COL2A1 and ACAN in OA chondrocytes. This is achieved at least in part by inhibiting NF-kB activation in human OA chondrocytes *in vitro*. Thus, with this unique profile of actions HLM may prove to be a potentially attractive and new therapeutic/preventive agent for OA and assist in the recovery from cartilage based sports injuries.

## Competing interests

Product is "intended" to be commercial (Vital g-Netics, LLC) but is indeed not yet released for commercial purposes. This was an exploratory study for which the HLM was provided by Vital g-Netics. No other input or support was provided or sought from the company.

## Authors' contributions

NA carried out the experimental work, collection, interpretation and manuscript drafting. MJSM helped with data interpretation and drafting of the manuscript. TMH conceived of the study, its design, coordinated, data interpretation and manuscript drafting. All authors have read and approved the final manuscript.

## Pre-publication history

The pre-publication history for this paper can be accessed here:

http://www.biomedcentral.com/1472-6882/11/66/prepub
